# The Empirical Power of Rare Variant Association Methods: Results from Sanger Sequencing in 1,998 Individuals

**DOI:** 10.1371/journal.pgen.1002496

**Published:** 2012-02-02

**Authors:** Martin Ladouceur, Zari Dastani, Yurii S. Aulchenko, Celia M. T. Greenwood, J. Brent Richards

**Affiliations:** 1 Department of Human Genetics, McGill University, Montreal, Canada; 2 Lady Davis Institute for Medical Research, Jewish General Hospital, Montreal, Canada; 3 Department of Epidemiology, Biostatistics and Occupational Health, McGill University, Montreal, Canada; 4 Department of Epidemiology, Erasmus MC, Rotterdam, The Netherlands; 5 Institute of Cytology and Genetics SD RAS, Novosibirsk, Russia; 6 Department of Oncology, McGill University, Montreal, Canada; 7 Department of Medicine, Jewish General Hospital, McGill University, Montreal, Canada; 8 Twin Research and Genetic Epidemiology, King's College London, London, United Kingdom; Wellcome Trust Sanger Institute, United Kingdom

## Abstract

The role of rare genetic variation in the etiology of complex disease remains unclear. However, the development of next-generation sequencing technologies offers the experimental opportunity to address this question. Several novel statistical methodologies have been recently proposed to assess the contribution of rare variation to complex disease etiology. Nevertheless, no empirical estimates comparing their relative power are available. We therefore assessed the parameters that influence their statistical power in 1,998 individuals Sanger-sequenced at seven genes by modeling different distributions of effect, proportions of causal variants, and direction of the associations (deleterious, protective, or both) in simulated continuous trait and case/control phenotypes. Our results demonstrate that the power of recently proposed statistical methods depend strongly on the underlying hypotheses concerning the relationship of phenotypes with each of these three factors. No method demonstrates consistently acceptable power despite this large sample size, and the performance of each method depends upon the underlying assumption of the relationship between rare variants and complex traits. Sensitivity analyses are therefore recommended to compare the stability of the results arising from different methods, and promising results should be replicated using the same method in an independent sample. These findings provide guidance in the analysis and interpretation of the role of rare base-pair variation in the etiology of complex traits and diseases.

## Author Summary

There is now evidence that rare variants can contribute to the etiology of complex disease. Next generation sequencing technologies have enabled their detection in large cohorts, and new statistical methods have been proposed to ascertain their association with complex diseases and traits in order to improve power over single-marker analysis. Each of these new methods assumes a particular nature of the relationship between rare variants and complex disease, yet these hypotheses have been largely unverified. Therefore we sought to compare the power of commonly used and novel statistical methods for rare variants using Sanger sequencing data from 1,998 individuals sequenced at 7 genes by simulating several phenotypes under models spanning a spectrum of the common hypotheses concerning such associations. While all methods perform reasonably well under their own model-specific hypotheses, no single method gives consistently acceptable power when these hypotheses are violated. Unlike GWAS, wherein all variants can often be tested using the same method across the entire genome, the analysis and interpretation of sequencing studies will therefore be considerably more challenging.

## Introduction

There is growing evidence that rare variants contribute to the etiology of complex diseases [1], [2], [3], [4]. A striking difference in the distributions of the odds ratios (ORs) for common and rare variants has been illustrated in a wide range of recent publications, favoring higher ORs for some rare variants (reviewed elsewhere [5], [6], [7]). As well, it has been demonstrated that rare coding variants associated with complex traits are sometimes causal through amino acid substitution [3], [8], [9]. For these reasons, rare variants hold promise as a source of heritability which is not explained by common base-pair variants.

Identifying rare variants associated with disease requires large sample sizes since few individuals harbor such polymorphisms. In addition, for rare variants, the power of single-marker tests, such as those performed by genome-wide association studies (GWAS), is poor. Development of alternative methods is thus essential. Over the past two years, a growing body of methods [2], [10], [11], [12], [13], [14], [15], [16], [17], [18], [19], [20] seeking to overcome this limitation has emerged. These methods generally employ three main strategies: collapsing markers across a region, weighting and/or prioritizing markers, and distribution-based approaches.

Li and Leal [20], for example, proposed a method to collapse rare variants across a region. This and other collapsing methods are based upon the hypothesis that low-frequency variants are rare, but in aggregate, they may be common enough to account for variation in common traits. Under such models, it is assumed that the probability of being diseased increases with the number of rare minor alleles. However, this might not always be the case [21]. Weighting methods assign more importance to alleles based on many possible criteria, such as minor allele frequency (MAF) in the control population [17], or possible alterations in protein function, including measures produced by SIFT and Polyphen2 [11], [22]. More recently, methods examining changes in distributions associated with rare variants [2], [23] have been proposed. Liu and Leal [2] based their novel method on multi-locus genotypic configurations, where each unique pattern of genotypes is tabulated, and the associated risk of disease for each configuration is modeled using a mixture distribution. Liu and Leal refer to their method as a kernel-based approach (KBAC), since part of the mixture distribution is modeled by nonparametric kernel density estimation. Neale et al. [23] showed that a test of association can be based on binomial over-dispersion of variance, conditional on the number of rare variants present in a region. Another innovative and flexible method has been developed by Wu et al [24]. These authors proposed the sequence kernel association test (SKAT), a supervised, flexible, and computationally efficient regression model (with the possibility of adjusting for covariates), to test the association between rare and common variants and traits or disease status. SKAT is similar to a classical mixed model, and is based on a score test for non-zero variance associated with the effects of all the rare variants under consideration.

These recently proposed models have often relied upon unverifiable (and sometimes unnecessary) hypotheses in order to simulate sequence data. Certainly, simulation of large sets of sequence data is a complex task and depends on hypotheses concerning the evolution of human genomic regions. The validity of any particular set of evolutionary hypotheses is unlikely to be consistently true across the [4] genome, as each gene demonstrates a large variance in these parameters [25], [26].

The performance of these newly proposed models using real sequence data in a large sample has not been independently evaluated. We therefore tested the power of commonly-used statistical methods designed to assess the impact of rare variants on continuous and dichotomous traits in 1,998 individuals Sanger-sequenced at seven genes. We employed a variety of possible relationships between genotype and phenotype in order to fully investigate the performance of such models under different realistic scenarios.

We selectively chose some of the recently proposed statistical methods for rare variant association. These included: collapsing methods (with and without a variable minor allele frequency [MAF] threshold for defining rare variants), a weighting method (which assigns weights variants inversely proportional to their MAF), a variance-based approach [2], [11], [17], as well as a regression method using the Kernel association test (SKAT) [24]. We used the software provided by [11] to implement the collapsing and weighting methods. Four models were first investigated: a collapsing method using a threshold of 1% (T1) and 5% (T5), a weighted approach (WE), and a variable-threshold approach (VT) (see http://genetics.bwh.harvard.edu/rare_variants). (Note that while the WE method was implemented by [11], the model was proposed by Madsen and Browning [17]). In addition, we developed an approach for detection of rare-variant association with continuous traits that was inspired by KBAC [2], that we call “weighted outlier detection” (WOD). Two different MAF thresholds were applied to this new WOD method at 1% (WOD1) and 5% (WOD5) (see Text S1 for details). The last method we tested is the regression model (SKAT) developed by [24]. The relative power of each of these methods was then compared assuming different possible relationships between rare variants and continuous traits or disease status.

## Results

We evaluated the comparative power of recently proposed rare variant association methods using Sanger sequencing data from 1,998 individuals.

### Control simulations

The first set of simulations, which are designed to act as positive and negative controls for each of the methods tested, assesses potential relationships between rare variants and continuous traits under the relevant hypotheses made in several models. Scenario 1 is a “null model”, which serves as a negative control. Scenario 2 acts as a positive control for all collapsing models. Scenario 3 depicts a mixture of rare and common variants. Scenario 4 is a positive control for SKAT and WOD, which are designed to perform well under a mixture of protective and deleterious variants. Scenarios 5 and 6 are positive controls for WE. Details for these six different scenarios are found in Table 1. (See Text S1 for additional information).

**Table 1:** Summary of phenotype simulations and hypotheses.

	Condition to be selected as a causal rare variants	Assumption	Number of causal variants	Mean effect size if carrying allele from a causal variant
**Scenario 1** **Null**	NONE	No association	NA	0
**Scenario 2** **Positive control for collapsing models**	MAF<0.01	At least one causal rare implies deleterious	at least one rare	−1.64
**Scenario 3** **Mixture of rare and common SNPs**	MAF<0.01	Causal SNPs are deleterious	4 rare and 4 common	−1.64 or −0.07
**Scenario 4** **Positive control for SKAT and WOD: Mixture of protective and deleterious SNP**	MAF<0.01	15% causal SNPs:7.5% deleterious, 7.5% protective	15%	−1.64 or +1.64
**Scenario 5** **Positive control for Weighting with sampling 1/MAF**	No restriction	Causal SNPs are deleterious, sampling with probability 1/MAF	10% of rare SNPs	Largest effect: −2.5Effect proportional to 1/MAF
**Scenario 6** **Positive control for Weighting with uniform sampling**	No restriction	idem 9, sampling of causal SNP is uniform	10% of all SNPs	Largest effect: −2.5Effect proportional to 1/MAF

**Figure 1:**
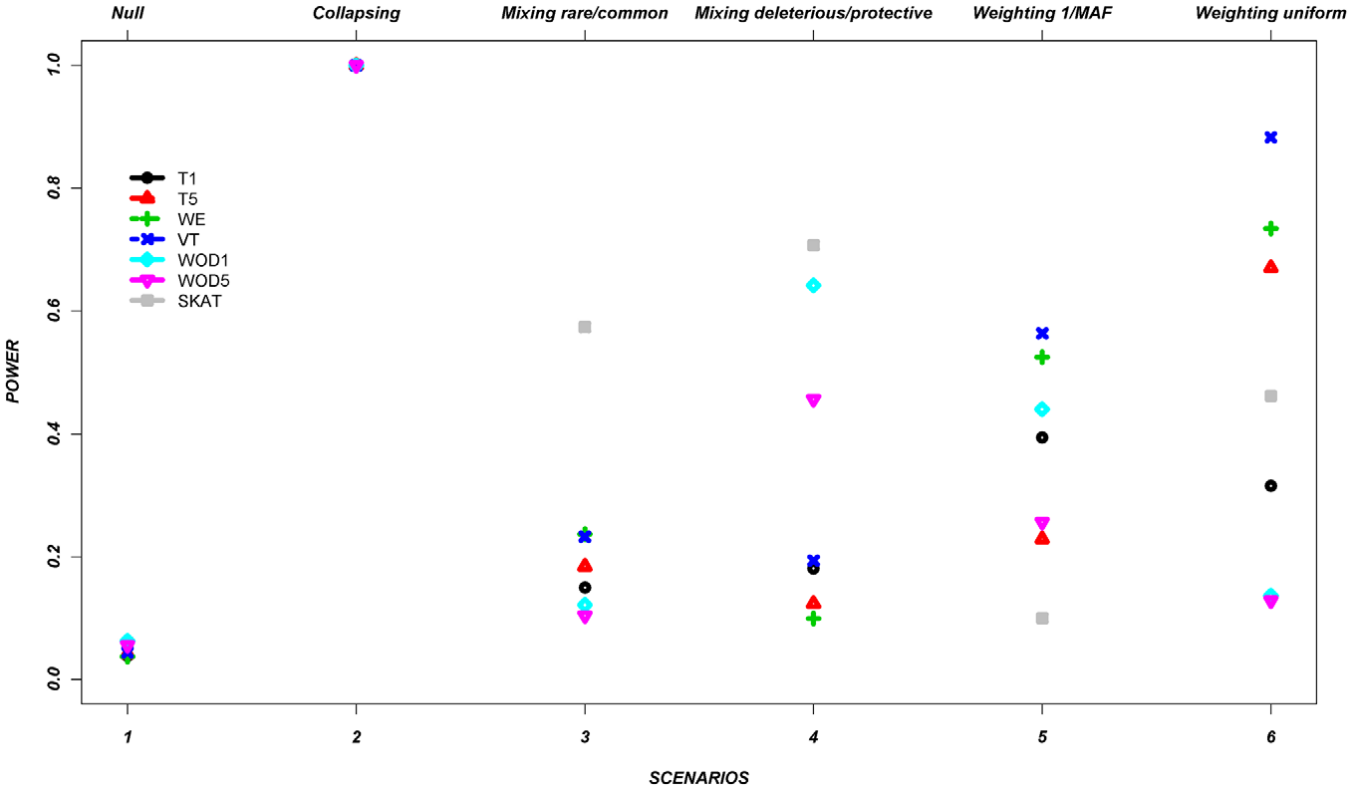
Power across all methods, per scenario, as described in **Table 1**, for the average across the seven genes. Footnote: Note that in some scenarios, different methods overlap. This is the case for scenario 1 and 2, where all methods give similar power.

Figure 1 shows the average power of each method based on this control set of simulations from all seven genes (Table 2 and Table 3). The power is around 5% in the null scenario, as expected, where no associations were assumed between the variants and continuous trait. On the other hand, Scenario 2, referred to as a positive control for the collapsing design, demonstrates power of 100%. It was expected that this latter scenario would lead to very high power, since the simulation assumed that the phenotypes were always altered if the individual carried at least one rare allele, such as would be expected with a highly penetrant allele.

**Table 2:** Description of the seven genes.

	Total number of variants in the gene	Number of rare variants (MAF<1%)	Median MAF	Mean percentage of missing genotype per variant	Coding length (Base pairs)
**Gene 1**	49	42	2.50E-04	4.9	2002
**Gene 2**	103	90	2.54E-04	3.9	4094
**Gene 3**	29	27	2.52E-04	11.2	1239
**Gene 4**	64	54	5.08E-04	3.9	1638
**Gene 5**	68	62	2.54E-04	2.9	1963
**Gene 6**	67	54	5.08E-04	4.1	2901
**Gene 7**	128	105	5.01E-04	7.2	1500

**Table 3:** Description of the count of rare variants per gene.

	Number of individuals with different counts of rare variants					
	0	1	2	3	4	5
**Gene 1**	1882	112	4	0	0	0
**Gene 2**	1707	223	34	4	1	0
**Gene 3**	1905	74	2	0	0	0
**Gene 4**	1719	240	9	0	0	0
**Gene 5**	1771	176	20	2	0	0
**Gene 6**	1735	223	10	0	1	0
**Gene 7**	1709	262	24	2	0	1

In the remaining scenarios, it is striking that all methods have relatively poor power under most hypotheses, even though our simulation design included large shifts in the mean phenotype in a large number of individuals. Almost all of these scenarios show power less than 50% in the majority of the methods. In scenario 3, the addition of common causal variants to the presence of rare causal variants did not improve the power, except for the SKAT method which demonstrates its advantage when combining common and rare variants. In Scenario 4, where bidirectional causal variants are present, only WOD1 and SKAT have power above 50%.

Scenarios 5 and 6 test performance when rarer variants have stronger effects. While the VT method marginally outperforms the WE method in these scenarios, the WE method improves considerably when compared to the other scenarios where no relationship was assumed between MAF and effect.

These results demonstrate that all methods perform well under their intended hypothesized relationship between rare variants and phenotypes, but their power can vary largely when there is departure from this main hypothesis.

We next assessed to what extent power is influenced by the effect size and proportion of causal rare variants. In the next set of simulations, we varied these two parameters to explore more systematically how much they influence the strength of the signal between genes and complex traits. The proportions of causal variants varied from 10, 15, 20, and 30% of all rare variants, where the causal variants were chosen at random from the polymorphisms that had low frequency (i.e., MAF≤1%). We assumed seven possible values for the mean effects: 0.5, 0.75, 1, 1.25, 1.5, 2, and 2.5 standard deviations.

### Systematic set of simulations (second set)

The combination of seven effects, four proportions of causal variants associated with the trait, and seven genes, leads to 196 scenarios. In these scenarios, we first analyzed the results of each scenario using single-marker tests, and then next applied the seven rare variant methods (T1, T5, VT, WE, WOD1, WOD5, and SKAT) for gene-level analysis. Next, we applied all seven methods to dichotomous traits, created by selecting from the extreme quarters of the continuous trait distribution.

### Single-marker tests for rare variants

**Figure 2:**
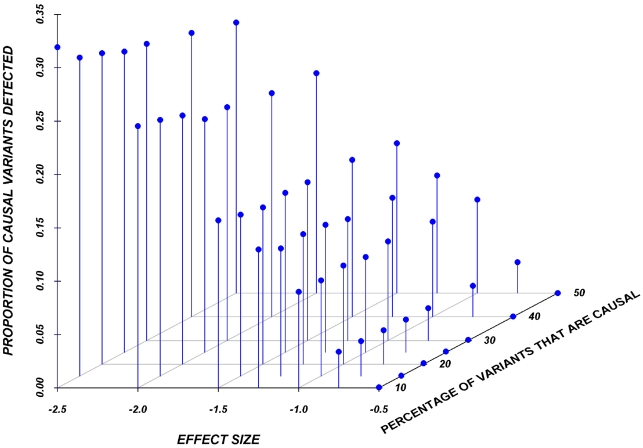
Proportion of causal variants reaching significance as a function of the average effect and proportion of causal variants on average in a gene, employing a SNP-by-SNP analysis.

Results here are restricted to analysis only of the assigned causal variants, and we report the proportion of these causal variants that reach statistical significance, after adjustment for multiple-testing, using a Bonferroni correction. Figure 2 shows the relationship between the proportion of causal variants assigned and their effect, averaged across all seven genes. Notably, but as expected, single-marker tests cannot identify more than 20% of the causal variants, even when effects are as large as 1.5 standard deviations. Power is particularly poor when the effect is 0.5 or 0.75 standard deviations.

### Systematic simulations for continuous traits

**Figure 3:**
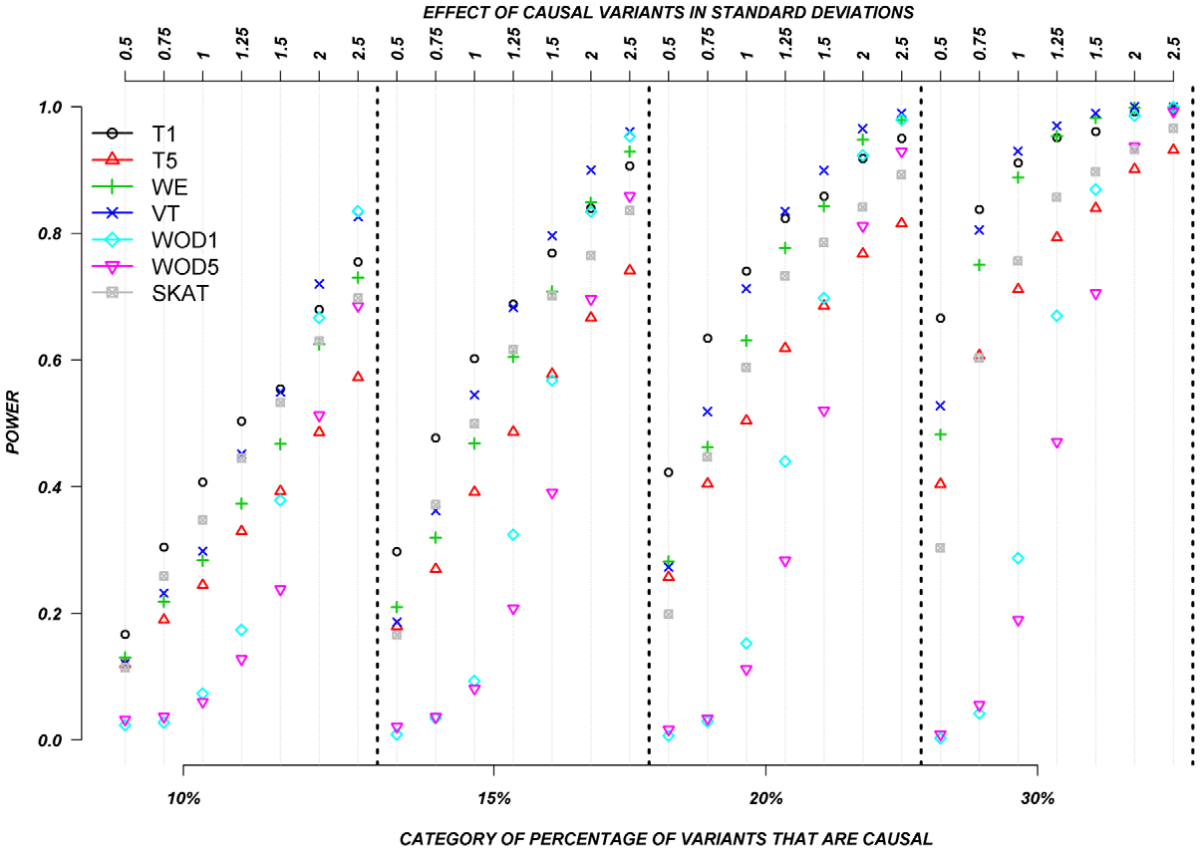
Continuous traits: Relationship between effect size, proportion of causal variants, and power. All causal variants have a deleterious effect. Each box corresponds to a different proportion of causal variants involved in the relationship between rare variants and continuous traits (from left to right, 10, 15, 20 and 30%). On the x-axis, effect sizes are in standard deviations and correspond to the absolute value of the average size effect.

Figure 3 shows the relationship between power, effect size, and proportion of causal variants associated with a continuous trait, averaged across all seven genes. Each dot represents the power of a given method ordered by average effect (ranging from 0.5 to 2.5 standard deviations) within each bin. Each bin represents the proportion of causal variants (ranging from 10 to 30%). Each of these 28 scenarios (7 different effect times 4 proportions of causal variants) can also be expressed in terms of proportion of variance explained, as seen in Table 4. These values indicate how much variability in the trait each simulated model explains. It is clear that none of the proposed methods have strong power to detect any gene when rare causal variants have small-to-moderate effects (less than 1.25 standard deviations). For most methods, effects of 1.5 standard deviations are needed to have reasonable power to detect an association. The power for most methods was less than 60%. Furthermore, our WOD method is not well powered for small-to-moderate effects, but is comparable to other methods when the effects are larger. Power tends to increase as the proportion of causal variant increases, mainly because there are more causal variants that can possibly influence phenotype. Note also that WOD does not accommodate covariates but that it remains possible to incorporate covariates into the phenotype by using residuals.

**Table 4:** Proportion of variance explained by rare variants.

	0.5 SD	0.75 SD	1.0 SD	1.25 SD	1.5 SD	2.0 SD	2.5 SD
**10% of causal**	0.005	0.012	0.022	0.034	0.049	0.086	0.135
**15% of causal**	0.008	0.018	0.032	0.051	0.073	0.129	0.202
**20% of causal**	0.011	0.024	0.043	0.067	0.097	0.172	0.269
**30% of causal**	0.016	0.036	0.064	0.100	0.144	0.256	0.400

Collapsing methods do not perform well when effects are small or moderate (<1.5 standard deviations). The only situation where the power was greater than 75% is when between 15% and 30% of the rare variants are causal, and effects are moderate-to-large (Figure 3). The SKAT method seemed to perform as well as most methods for smaller proportion of causal variants, but underperforms as the proportion of causal variants increases.

**Figure 4:**
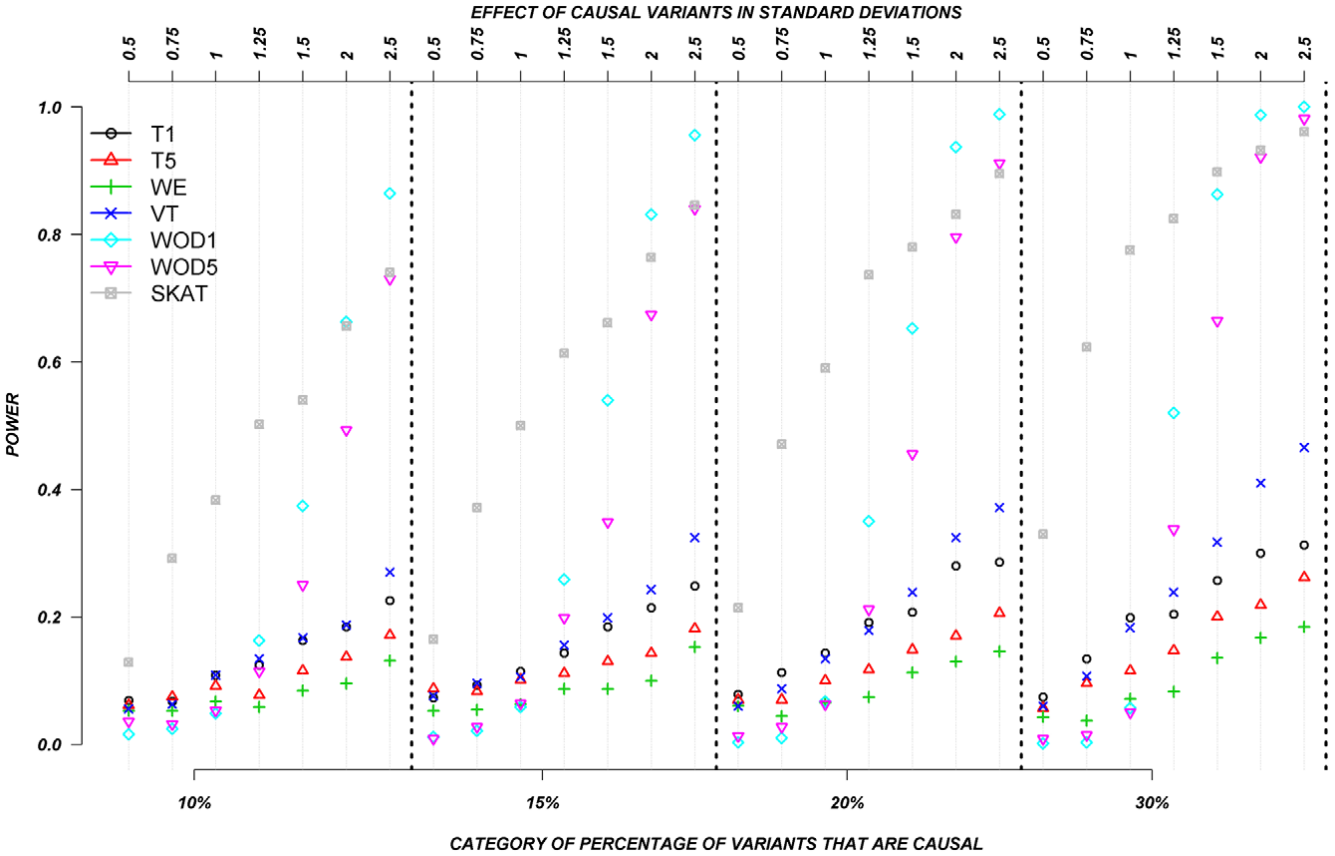
Continuous traits: Relationship between effect size, proportion of causal variants, and power, when causal variants are a mixture of protective and deleterious effects. Each box corresponds to a different proportion of causal variants involved in the relationship between rare variants and continuous traits (from left to right, 10, 15, 20 and 30%). On the x-axis, effect sizes are in standard deviations and correspond to the absolute value of the average size effect.

We also evaluated the power of the rare variant methods when rare variants are assigned to have either deleterious or protective effects (Figure 4). In this set, we permitted half the causal variants to be deleterious and half to be protective. Again, the assigned absolute effects ranged from 0.5 to 2.5 standard deviations and the proportion of causal variants ranged from 10–30%. Figure 4 clearly shows the substantial advantage of SKAT and our distribution-based approach (WOD) to detect effects in this context. In the case of WOD, however, this advantage is limited to effects of more than 1.5 standard deviations. SKAT does perform better than WOD when the mean effect is small, but this advantage tends to disappear for larger effects, e.g., over 2.0 SD. When individuals carrying causal alleles have phenotypes shifted by less than 1.25 standard deviation, all methods, except SKAT performed equally poorly. In these situations SKAT provides clearly improved power, but absolute power remains relatively low. These results clearly show the important contribution of methods that can account for mixture of protective and deleterious variants within a gene.

### Systematic simulations for dichotomous traits

In order to assess the performance of these methods for dichotomous traits, we selected 500 cases and 500 controls from the extreme 25^th^ percentiles of the continuous trait distributions. This design therefore tests power of rare variant methods for sampling designs targeting more extremes of the distribution.

**Figure 5:**
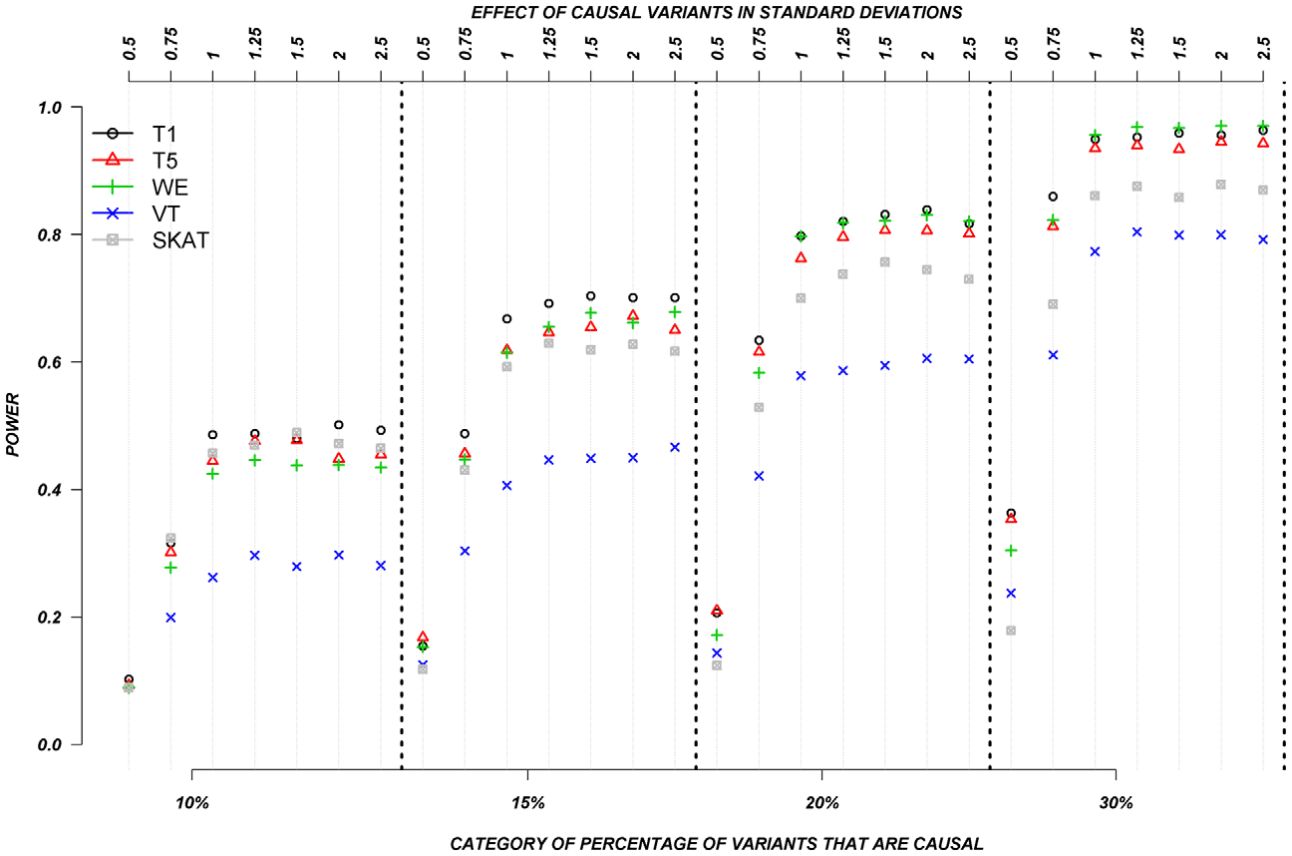
Dichotomous traits: Relationship between effect size, proportion of causal variants, and power, when causal variants only have a deleterious effect. Each box corresponds to a different proportion of causal variants involved in the relationship between rare variants and continuous traits (from left to right, 10, 15, 20 and 30%). On the x-axis, effect sizes are in standard deviations and correspond to the absolute value of the average size effect.

Figure 5 shows the relationship between power, effects, and proportion of causal variants associated with a dichotomous trait, when causal rare variants only increase risk of disease, averaged across all seven genes. Note that WOD was not designed for dichotomous traits, so results from this model are not presented in this section.

Again, power increases as the proportion of causal variants increases, and power remains low for smaller effects. In this particular case-control design, VT appears to have the lowest power compared to all other methods. The remaining methods, T1, T5, WE, and SKAT have power estimates that are in a similar range, but power from T1, T5, and WE seemed to outperform SKAT as the proportion of causal variants increases. Interestingly, power does not seem to be as strongly influenced by the magnitude of the effect, as is it for continuous trait results. This can be explain by the fact that when the effect is one SD away from the mean, on average, over 90% of the individuals that are carrying a causal allele will have their phenotype shifted and be classified as cases. In other words, between effects of 1 to 2.5 SD, there is not a large difference in the number of shifted individuals that are correctly classified as cases.

**Figure 6:**
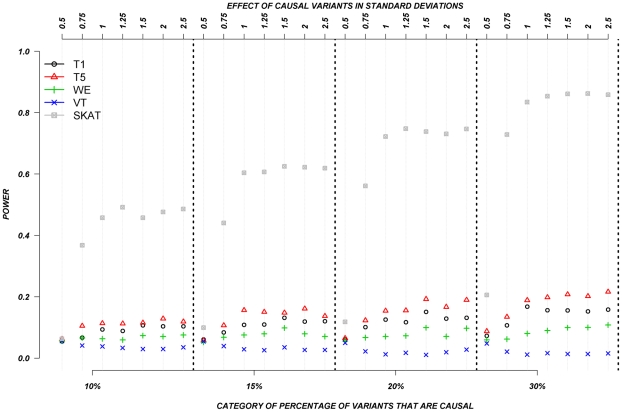
Dichotomous traits: Relationship between effect size, proportion of causal variants, and power, when causal variants are a mixture of protective and deleterious effects. Each box corresponds to a different proportion of causal variants involved in the relationship between rare variants and continuous traits (from left to right, 10, 15, 20 and 30%). On the x-axis, effect sizes are in standard deviations and correspond to the absolute value of the average size effect.

The power was low for almost all methods when causal variants could be either deleterious or protective––as was observed for continuous traits. Figure 6 shows the relationship between power, effects, and proportion of causal variants associated with a dichotomous trait, when causal variants are deleterious, or protective, averaged across all seven genes. Power increases as the proportion of causal variants increases, and we also observe the “plateau” pattern described in the previous paragraph. Methods such as T1, T5, and WE that are not designed for a mixture of deleterious and protective effects have poor power to detect any association between genes and dichotomous traits. SKAT clearly outperforms the other methods under these circumstances.

## Discussion

While many large-scale sequencing studies are now underway to identify rare variants associated with complex diseases and traits, our results demonstrate that assessing the association between rare variants and complex disease is a challenging task. Standard single-marker association methods exhibit low power and the power of the statistical methods tailored for rare variants varies tremendously depending on the true nature of the relationship between the rare genetic variants and the phenotype. These findings provide guidance in the design, analysis and interpretation of sequencing studies for complex disease.

As it is still unknown how rare variants influence complex disease, we have simulated several phenotypes under models spanning a spectrum of the common hypotheses concerning such associations. It is likely that the nature of the relationship between rare variants and a phenotype varies from gene-to-gene. Our findings suggest that no single method gives consistently acceptable power across the range of these relationships, even in a large sample size. Analysis using different methods clearly imposes an additional multiple testing burden, which cannot be easily addressed. One, though somewhat cumbersome, way to solve this problem would be by derivation of empirical P-values taking into account the variety of methods tested. Another, more straightforward, approach would be to undertake replication in an independent sample, using the method which demonstrated best results at the discovery stage.

In this paper, we have also developed a new method conceptually based on Liu and Leal's KBAC method [2] to detect the association between rare variants and quantitative traits. Our extension of [2] is implemented in R and is available from the authors. We have also developed a simulation framework to compare all major novel statistical methods to identify the contribution of rare variants to continuous phenotypes under identical conditions. Our new approach performs poorly if all rare variants act in the same direction, but performs well when variants can either increase or decrease phenotype and have large effect. We note that the presence of randomly assigned rare variants of smaller effect in size, all tests have a distribution of test statistics that follows the null distribution (see Text S1).

Collapsing methods demonstrate increasing power when the trait varies with an increasing number of rare alleles. However, examples exist where protective and deleterious rare alleles are present in a gene [21], and in such situations, collapsing methods do not perform well. On the other hand, SKAT and WOD performed extremely well compared to other methods in the continuous traits scenarios, and dichotomous traits (SKAT only) scenarios, respectively. SKAT in particular, was the only method that performed well for dichotomous traits when variants could be protective or deleterious. Methods like WE that assign more weight to rarer alleles are promising, but only if the gene harbors several causal variants whose effects are each inversely proportional to their MAF. However, we note that the VT method still outperforms WE even when employing this assumption.

Our study also provides empirical data to judge the value of dichotomizing a continuous trait and sequencing only its extremes. While our design included the extreme quarters of the distribution, thereby eliminating the need to sequence half the study population and consequently reducing sequencing costs substantially, we note that power was similar to that derived from the entire distribution particularly only when the proportion of causal variants was high and the effect sizes moderate. Nonetheless, sampling of the extremes remains an attractive study design, particularly if the sampled population is large and a more extreme sub-population is selected.

Methods have been proposed to weight the relative importance of rare variants based on various parameters including their estimated deleterious effect on protein function [17], [27]. For example, the incorporation of estimated functional information, such as the potential effect of an amino acid change as estimated by Polyphen or SIFT, might improve power. However, these scores have been criticized for their high level of misclassification [22]. Moreover, functional prediction is more challenging when the variants are non-coding.

The spectrum and frequencies of rare genetic variants are known to depend on ancestry and age of the population studied [28]. In this work, we have assumed that our sample consists of a homogeneous population without stratification into population subgroups. All the methods that we have examined could find false associations if population sub-strata existed and were associated with the phenotype, therefore particular attention must be paid to population structure when designing rare variant studies.

One of the strengths of our study is the use of Sanger sequencing data, rather than simulated genotyping data. We have been able to avoid the simulation of such data by using fully Sanger-sequenced data on nearly 2,000 individuals at seven genes. Therefore, no genotypic hypotheses were made to generate the sequence data. Furthermore, the sample size employed is among the largest sequenced datasets in the world at present. Despite the fact that gene 3 had more missing data and fewer variants, we note that the power results derived from this gene are similar to all other genes.

We note that our simulations assumed no additive effects when an individual carries multiple rare variants. However, we note that very few individuals carry 2 or more rare variants (Table 3). In addition, we assumed that rare variant effects take precedence over common variant effects.

In light of our results, we recommend that single-marker tests should not be used alone when rare variants are present and are assumed to have small-to-moderate effects on the trait of interest. On the other hand, as power across all novel rare variants methods is generally low, the potential for identifying rare variant associations using gene-based analysis strategies requires improvement. Ideally, the true underlying nature of the association between the gene and the phenotype should determine the choice of statistical method, however, this relationship is almost always unknown. Therefore, performing sensitivity analyses, i.e., assessing different methods that perform differently under various conditions might be a helpful option in order to interpret the results. Furthermore we suggest that if one method identifies a gene of interest that replication of this result should be performed in an independent sample using the same statistical method. All methods seemed to perform adequately under their specific model hypotheses, but do not perform as well when these hypotheses are violated.

In the next few years, advances in sequencing technology will enable the production of large quantities of sequence data on large numbers of individuals, allowing for the cost-effective identification of rare variants. These data will enable researchers to investigate the role that rare variants play in disease etiology, in addition to uncovering functional variants. Our results may provide guidance in the planning, analysis and interpretation of these large-scale initiatives.

## Materials and Methods

### Ethics statement

The work described in this manuscript represents a re-use of data and no new human interventions were conducted. No additional IRB approvals were sought for this specific portion of the work. The Committee on Ethics in Clinical Research, CHUV, Lausanne University, Lausanne, Switzerland approved the original protocols for sample collection.

### Study sample

The subjects used in this paper are a subset of the CoLaus study, a population-based study of 6,188 Lausanne residents aged 35 to 75 years [29].

### Sanger sequencing data

Sanger sequence data for the exons and flanking regions of seven genes including *PLA2G7* from 1,998 individuals were provided by GlaxoSmithKline (GSK). Methods for performing the sequencing for the *PLA2G7* gene and the additional 6 genes have been described [30]. The identity of the remaining genes was not disclosed for proprietary reasons. Sanger sequencing has a low error rate and is considered a gold-standard for comparison to high-throughput sequencing studies [31], [32]. For simplicity, and since rare variants are not expected to be in high linkage disequilibrium (LD) with surrounding variants, we imputed the missing values of each rare variant independently from others based on the computed MAF. The percentage of missing genotypes per variant in a gene ranged from 3% to 11%, with an average of 5.5% individual missing genotype information per variant, across all genes (Table 2). All non-polymorphic base-pair markers were removed from the sequence data.

All seven genes contained both rare and common variants: the number of polymorphic variants ranged from 29 to 128, and the proportion of variants with a MAF≤1% ranged from 81% to 93%. The majority of these variants were extremely rare, with an average of 55% of all variants across all genes being singletons. Table 2 and Table 3 describe the allelic frequencies, and rare variant distribution of all seven genes. We used these known genotypes combined with phenotype simulations to compare several commonly-used and novel statistical methods developed for rare variants and continuous phenotypes.

### Parameters influencing rare variant associations with complex traits

We developed two simulation sets to illustrate the power of a variety of commonly-held hypotheses about the possible effects of rare variants on complex traits. In the first set, we tested collapsing and weighting designs and a range of general concepts about the potential role of rare variants, whereas in the second set, we varied the effect and the proportion of causal variants in across a grid of values.

### Control simulation sets

We proposed different phenotype simulation scenarios to explore popular hypotheses regarding the mechanism by which rare variants could influence complex disorders, namely (a) the assumption that risk of disease increases with more rare alleles (collapsing design), (b) the assumption that the magnitude of the effect depends on MAF (such as equation (1) in [17] for the weighting design), and (c) performance when a mixture of deleterious and protective causal rare variants influences phenotypes (Table 1). Here we describe the motivation behind our choice of scenarios. Scenario 1, the null model, contains no causal variants. Scenario 2 assumes that any rare variant increases the risk of disease, which reflects the hypothesis underlying many of the proposed statistical methods. Scenario 3 investigates a mixture of common and rare causal variants, Scenario 4 investigates a mixture of deleterious and protective effects, and Scenarios 5 and 6 explore the assumption that variants with lower MAF have larger effect. In these cases, the effects were derived from equation 1 in [17].

In our simulation of phenotypes, the following rules were applied in all scenarios. We assumed that all non-carriers of a causal allele (deleterious or protective) variant have a normally distributed trait with mean zero and variance of one, using a standard normal random variable. When one or more *common* variant(s) is/are assumed to have a deleterious effect, and an individual is carrying at least one of these causal alleles, we randomly drew a phenotypic value from a normal distribution having a mean of −0.07 and a standard deviation of 1.01, which allows for an effect typically identified in GWAS studies of continuous traits [33], [34], [35]. When a rare variant is assumed to be deleterious, carriers of at least one rare causal allele had a phenotypic value randomly sampled from a normal distribution with mean at −1.64, and standard deviation of 0.2. Relative to the phenotype distribution of individuals with no causal variants, these means correspond to the bottom 5% of the distribution. Similarly, to model protective effects of a rare variant, the assigned effect was normal with mean +1.64 and a standard deviation of 0.2. Such effects for rare variants have been observed in the lipid literature [36], [37].

Deleterious variants were randomly sampled from the pool of variants for each simulation. Rare variants were defined as those having a MAF 1%, and common variants were defined as >1%. While other thresholds can be used, GWAS have often used a 1% threshold to define rare variants [35]. We allowed all rare variants to be possibly causal, including singletons. Table 1 summarizes the parameters investigated.

By varying hypotheses about the sampling of causal variants and their effect, we created these 6 simulation scenarios. We randomly generated a set of 250 phenotypes per individual, per scenario, per gene. In each case, we randomly selected causal variants associated with the traits, and then randomly generated a set of phenotypes based on the corresponding parameters for each iteration.

### Systematic simulation sets

In our second series of simulations, we varied the proportion of causal rare variants and their average effect on the phenotype across a grid of values, i.e., where proportions (10, 15, 20, and 30%) of causal rare (MAF≤1%) variants were combined with values (0.5, 0.75, 1, 1.25, 1.5, 2, and 2.5 standard deviations) for the mean effects. We also report in Table 4 the proportion of variance explained by rare variants for each combination of proportion of causal rare variants and their effect. An individual carrying at least one rare causal allele has their phenotype value chosen randomly from a normal distribution with one of these seven means and with a standard deviation of 0.2. All 28 combinations between the proportion of causal (four values) and effect (seven values) were simulated for the seven genes. Two hundred and fifty sets of phenotypes were generated.

Multiple-testing was taken into account for single-marker test analyses, using a conservative approach with Bonferroni correction for the number of single-nucleotide polymorphisms (SNPs) tested. As for other rare variant methods, permutation was used to control for type-I error in all statistical methods. Alpha level was set to 0.05.

We also simulated dichotomous phenotypes by assuming selection from the extremes of a quantitative distribution. In each of the 196 scenarios presented above for continuous traits, we have defined cases as being the 500 individuals with the lowest continuous phenotypes, and the controls as being the 500 individuals with the highest continuous phenotypes. This study design allows direct comparison of the relative utility of sequencing only the extremes of a distribution, as compared to the entire distribution, which has considerable financial ramifications

## Supporting Information

Text S1 Details on the WOD method, additional details on how the phenotypes were simulated for each scenario, and QQ plots under the null hypothesis for all methods, and for genes 6 and 7.
(DOCX)
