# Supplementary material for: The Empirical Power of Rare Variant Association Methods: Results from Sanger Sequencing in 1,998 Individuals
Source: PLoS Genet. 2012 Feb 2;8(2):e1002496. doi: 10.1371/journal.pgen.1002496 (PMC3271058; doi:10.1371/journal.pgen.1002496)
Supplement: Text S1 — Details on the WOD method, additional details on how the phenotypes were simulated for each scenario, and QQ plots under the null hypothesis for all methods, and for genes 6 and 7. (DOCX) [file pgen.1002496.s001.docx]

**Supplement 1**.

**An outlier-based approach for continuous traits**

Methods that exploit the changes in distributions associated with rare variants are showing promise [1]. Liu and Leal [1] based their recently-published method for detecting rare-variant associations with disease on multi-locus genotypic configurations in a candidate region; each unique pattern of genotypes is tabulated. In their method, conceptually, risks of disease associated with each configuration are modeled with a mixture distribution, with one distribution for individuals not carrying influential variants, and another distribution for individuals with causal variants. The ‘normal’ distribution of risks is estimated from the subset of individuals who carry no rare variants. We will refer to such individuals as carrying a “null genotype configuration”.

For continuous phenotypes, we took a similar strategy and characterized the phenotypic distribution for individuals with no rare variants by a normal distribution with the mean and variance observed in this subset. For all who carry at least one rare variant, the absolute difference between the phenotype, y_i_, and the null-group mean, y_0_, is weighted by the two-tailed p-value arising from this normal distribution, and these differences are then summed across all individuals. Therefore, this approach detects variants that influence the phenotype in either direction and the statistic will be large when there is a cluster of phenotypic outliers. Permutations were used to evaluate significance.

**Scenario Details:**

**Scenario 1**

Scenario 1 is the null scenario, in which the whole cohort has a phenotypic value generated from a standardized normal distribution.

**Scenario 2**

This next scenario is based on the popular assumption that carrying at least one allele from any rare variant is sufficient to have a different phenotypic distribution than individuals that are carrying any allele from the rare variants. MAF of the potential deleterious rare variants are again defined as those with a MAF<0.01. Distribution of the phenotype for individuals carrying at least one deleterious allele is N(-1.64, 0.2).

**Scenario 3**

Scenario 3 combines the assumption that rare and common variants can be associated with a phenotype. Individuals that carry at least one rare causal allele will have a phenotypic value that is generated from a N(-1.64, 0.2), no matter whether they carry an allele from the common variant or not. Those who carry the causal common allele, but no allele from the deleterious causal rare variant, have a phenotypic value that is normally distributed from a standard normal, to which we subtract a random value from a N(-0.07, 0.01).

**Scenario 4**

In this scenario, 15% of rare variants are assumed to be causal, with the addition that half of the causal are deleterious, and the other half protective. Having both causal rare alleles has no impact on the phenotype.

**Scenario 5**

In this scenario, causal variants are sampled based on the inverse of their MAF. The proportion of causal variants is 10%. The effect of each variant is based on their MAF, with the variant having the lowest MAF receiving an effect of -2.5. The rest of the effect follows equation 1 in Madsen and Browing.

**Scenario 6**

This scenario is identical to Scenario 5, except that the sampling of the variant is uniform.

**QQ plots:**

NULL HYPOTHESIS:

Under the null hypothesis, we have selected 2 series of QQ plots, using phenotypes obtained from gene 6 and 7, but analyzed using gene 2 and 1, respectively. This yields a set of phenotypes which are randomly generated with respect to the genotypes, from which we observe that the signals generally follow the distribution of test statistics expected under the null. The set of QQ plots are those obtained under the null hypothesis of scenarios where effect = 0.5 SD, and proportion of causal variants = 10%.

Gene 6.

Figure S1


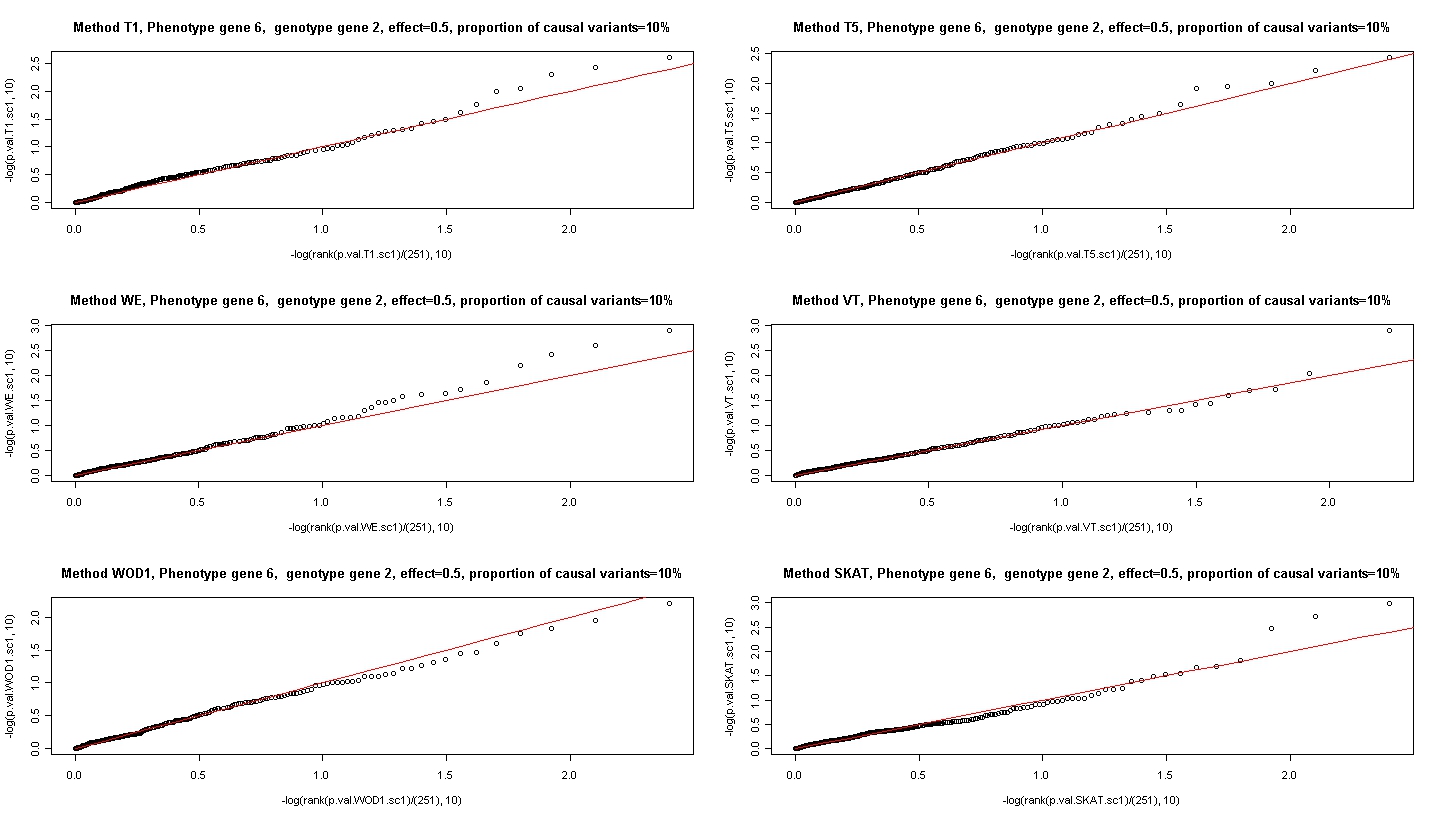


Gene 7.

Figure S2


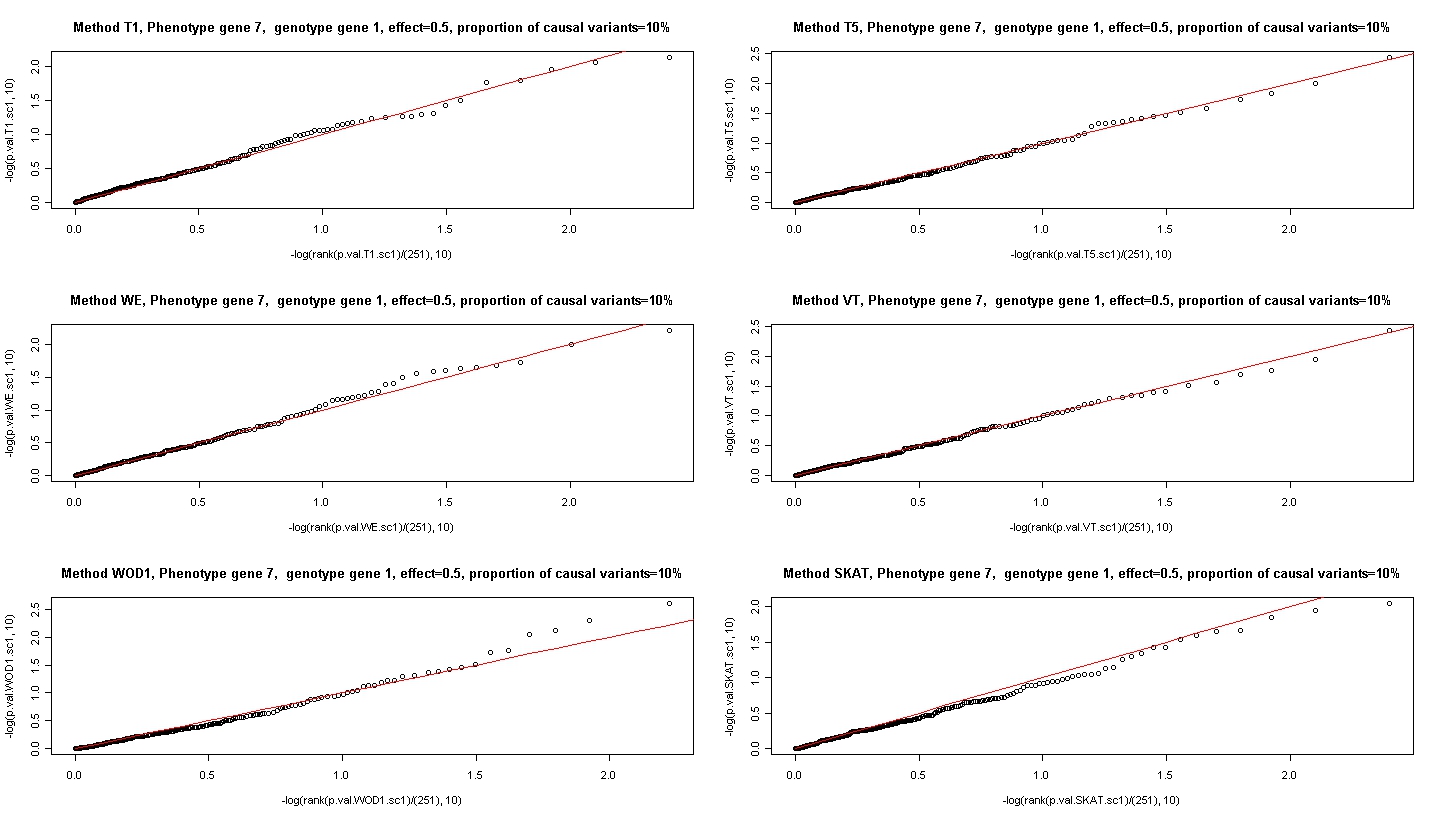


Reference

[1] Liu DJ, Leal SM (2010) A novel adaptive method for the analysis of next-generation sequencing data to detect complex trait associations with rare variants due to gene main effects and interactions. PLoS Genet 6: e1001156.
